# Balancing Innovation and Equity: A Successful Dynamic Between Private and Public Sectors Is Essential to Ensure True Pandemic Influenza Preparedness

**DOI:** 10.3390/vaccines13111078

**Published:** 2025-10-22

**Authors:** Lyn Morgan Marsden, Marie Mazur

**Affiliations:** 1 Sanofi SA, 69007 Lyon, France; 2 MARIMA LLC, Kennett Square, PA 19348, USA; marie-georges@mazur01.com

**Keywords:** pandemic influenza preparedness, seasonal influenza, vaccine manufacturers, public–private partnerships, equitable access, multilateral frameworks

## Abstract

The COVID-19 pandemic demonstrated both the transformative capacity of vaccine innovation and the persistent inequities that accompany emergency access, underscoring the critical need for stronger collaboration between global health governance and the vaccine industry. Influenza pandemics remain inevitable threats. The continued emergence of avian influenza strains such as H5N1 reinforces the necessity of robust preparedness. This perspective examines the underutilization of private sector vaccine manufacturers in current pandemic influenza frameworks and identifies three central areas where industry participation is indispensable: predictable vaccine demand through robust seasonal influenza programs, economic incentives that de-risk investments in research and development, and diversification of vaccine platforms to expand response capacity. In addition, regionalizing manufacturing, advancing collaborative regulatory models, and negotiating export waivers are presented as potential mechanisms to strengthen equity and supply security. The review highlights demand-based tiered pricing and Advance Purchase Agreements as practical tools to align commercial incentives with public health priorities. Furthermore, it makes the case for embedding private sector representation and knowledge into top-level decision-making and preparedness planning, ensuring investment in innovation is aligned with global health objectives. Ultimately, true pandemic influenza readiness depends on building a sustained seasonal influenza market, embedding private sector engagement into governance structures, and fostering mutual trust to ensure timely access and equitable protection for populations worldwide.

## 1. Introduction

The COVID-19 pandemic was a watershed moment for vaccine innovation and global health, demonstrating the transformative potential of public–private collaboration in vaccine development while exposing persistent inequities in access and delivery.

Influenza pandemics have killed, and will again kill, millions of people. It is estimated that the influenza pandemic of 1918 infected one-third of the world’s population and led to at least 50 million deaths before it subsided in 1920, killing more than eight times as many people as COVID-19 had killed—over 6.65 million—by 2022 [1,2]. We will discuss further in this perspective that influenza pandemic planning has advanced since the influenza A (H1N1) 2009 pandemic [3,4]; nonetheless, we argue that industry could and should be better utilized as a strategic partner in influenza preparedness frameworks, through being more efficiently solicited during the inter-pandemic period to ensure delivery of appropriate volumes of pandemic vaccine on time.

As the world faces the inevitability of future influenza pandemics, it is critical to strengthen the relationship between private sector vaccine manufacturers and global health governance. This perspective explores the drivers that motivate private sector engagement, the structural limitations that must be addressed, and some governance mechanisms required to ensure both innovation and equity in pandemic influenza preparedness. We summarize key themes and challenges at the interface of public health policy and vaccine industry engagement, highlighting strategic opportunities and offering recommendations for future practice. As such, the statements and proposals advanced in this perspective do not represent the results of primary research or formal consensus-generating processes but rather aim to stimulate further productive discussion and inform both policy and practice in this rapidly evolving field.

## 2. Drivers for Private Sector Engagement in Pandemic Influenza Preparedness

### 2.1. Predictable Seasonal Vaccine Demand Is Core to Pandemic Vaccine Readiness

The world’s pandemic influenza vaccine manufacturing capacity is primarily dictated by seasonal influenza vaccine volumes. The global demand for seasonal influenza vaccines is stable at approximately 760 million doses in 2022, with 97% (over 720 million doses) of those vaccines consumed by high and upper-middle income countries and 92% (around 700 million doses) consumed by countries in the regions of the Americas, Europe, and the Western Pacific [5]. The World Health Organization (WHO) last published its estimates for global production capacity of seasonal and pandemic influenza vaccines in 2023 [6], showing that there is influenza vaccine production capacity in all regions of the world, except the African region.

Seasonal influenza bulk vaccine production capacity remains relatively stable at 1.53 billion doses and pandemic capacity at 4.13 and 8.26 billion doses for moderate and best-case scenarios, respectively. Should the ongoing H5N1 Avian Influenza virus, which has spread throughout US dairy farms over the last 12 months, adapt for sustained human transmission and spark a pandemic, it is reasonable to assume that most H5N1 human vaccines matching the new H5N1 pandemic clade would require two doses—usually spaced apart by about three weeks—to achieve a protective immune response [7].

The WHO global influenza vaccine production estimate highlights three persistent challenges:

- Potential supply chain vulnerabilities due to dependence on embryonated eggs and ancillary supplies; hence the need to expand alternative/next-generation technologies.
- Limited manufacturing capacity in low- and middle-income countries, especially in Africa.
- Recommendations for a broader manufacturing footprint, including strengthened seasonal influenza programs.

While pandemic influenza planning has advanced since the last pandemic in 2009 (H1N1) [8], the COVID-19 pandemic response showed that the vaccine industry remains underutilized as a strategic partner in preparedness frameworks, as shown by a general lack of integration of industry expertise into public health planning, fragmented collaboration efforts, and regulatory barriers that limited the speed and effectiveness of vaccine rollouts [9].

As seen during the COVID-19 pandemic, technological advances alone do not lead to equitable access: by the end of 2021, when 75% of those in wealthy countries had received two doses of COVID-19 vaccines, fewer than 2% of people in some of the poorest countries had received a single dose [10].

Vaccine manufacturers possess specialized knowledge about production, logistics and innovation but they are often not actively involved in top-level public health decision-making, leading to less informed strategies and the risk of slower implementation of critical solutions during a crisis. Furthermore, to overcome the absence of market-driven predictability, a robust pandemic preparedness strategy requires clear economic incentives and effective risk mitigation to mobilize vaccine manufacturers. Thus, vaccine industry participation in pandemic influenza preparedness is underpinned by a combination of public health imperatives and economic incentives. Governments and international agencies recognize that the private sector’s agility, manufacturing capacity, and innovation pipelines are indispensable for rapid vaccine development and deployment [11,12], and most also concede that this is not a classical market and hence requires specific risk mitigation mechanisms.

Some high-income countries (HICs), e.g., US, Australia, U.K., several EU countries, and Japan are actively preparing for an influenza pandemic by building stockpiles of pre-pandemic vaccines and/or securing future pandemic vaccine supplies through Advance Purchase Agreements (APAs), which function as a reservation of strain-matched vaccine doses and manufacturing capacity from domestic and/or foreign vaccine manufacturers [13].

Stockpiling of pre-pandemic influenza vaccines primarily aims to protect frontline workers. These stockpiles are updated as new threats/strains emerge. Still, it is generally deemed unrealistic or not cost-effective to maintain a stockpile for the entire population of a country since this would require destruction at the date of product expiry.

As a result, APA is a mechanism used mainly by HIC governments to secure protection of the broader population as soon as possible upon declaration of a pandemic. However, most countries have no agreement with influenza vaccine suppliers, relying on their government’s potentially last-minute decision to purchase pandemic vaccine doses, either directly or through multilateral organizations, in the event of a pandemic. The downside of this tactic is the unavoidable waiting period prior to dose delivery, since manufacturers are bound to honor their APA commitments first, and as was seen during COVID-19, export issues can block fluid access.

### 2.2. A Healthy Seasonal Influenza Vaccine Market Dictates Pandemic Capacity

**Figure 1:**
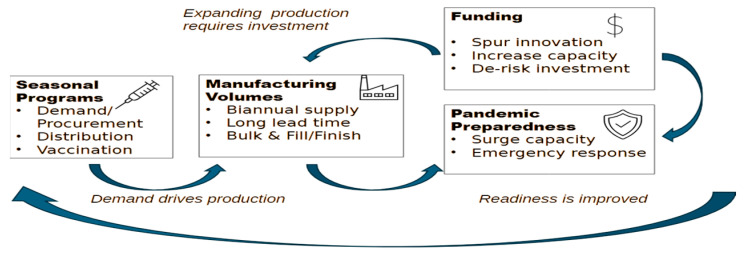
Influenza pandemic preparedness is anchored on seasonal influenza programs.

There is consensus between global public health partners and the vaccine industry that robust seasonal influenza vaccine coverage rates (VCRs) are a meaningful indicator of pandemic preparedness (see Figure 1). On one hand, seasonal vaccines drive manufacturing volumes and dimension the scale readily available to “switch” to pandemic manufacture, and on the other, a country’s ability to immunize adults is a key factor of success in a pandemic immunization program. The 2009 H1N1 influenza pandemic confirmed this to some extent since most countries with a robust seasonal influenza program were well-positioned to rollout access to the pandemic vaccination campaigns, especially in reaching vulnerable adults most at risk of severe effects [14]. Similarly, 12 months after the introduction of the COVID-19 vaccines, countries with an influenza vaccination program had reached an average vaccinated population of 47%, compared with 22% in countries without influenza programs [15] WHO recommends that all countries should consider implementing seasonal influenza programs [16], with a target of 75% coverage for the most vulnerable. As of 2022, 66% of WHO Member States (128/194) had a seasonal influenza vaccination policy, and more than half of the WHO Member States (100) recommended annual seasonal influenza vaccinations for WHO recommended priority groups (healthcare workers, people with chronic conditions, older adults, pregnant women, and other high-risk populations as relevant to their national context). Globally, the median vaccination coverage rates (reported by only 64 countries) varied by group: 37% for pregnant women, 55% for older adults, and 62% for healthcare workers [17]. Publications have reported seasonal influenza vaccination rates across multiple regions worldwide, but there is substantial variation in uptake depending on country, demographic group, and continent. Healthcare worker (HCW) vaccination rates by continent, reported in 2023, were 67.1% in the Americas, 51.3% in the Middle East, 48.7% in Oceania, 42.5% in Europe, 28.5% in Asia, and 6.5% in Africa; these values indicate that much remains to be done to move closer to the WHO target [18].

We believe that more resources should be available to turn policy into concrete actions on the ground. While a proportion of countries will quite understandably not prioritize influenza prevention due to other priorities, namely malaria, TB, and HIV, middle- and high-income countries can benefit economically from preventing hospitalization and/or lost workdays caused by influenza [19,20]. This is particularly pertinent in countries where non-communicable diseases (NCDs) present a significant burden, and/or where a significant proportion of the population is elderly.

Global policies are in place, including WHO‘s Immunization Agenda 2030, which calls out the importance of Life Course Immunization, and the Decade of Health Ageing (2021–2030). The complexity lies, at least in part, in prioritization, implementation, and technical support at the country level. Close coordination across governments, technical experts, and vaccine suppliers is key to building sustained programs step by step. Independent organizations like the Partnership for International Vaccine Initiatives (PIVI) unite these entities by applying a pragmatic approach, providing technical expertise and fostering best-practice sharing between countries [21]. Ultimately, demonstration of progress at a smaller scale (e.g., focusing on specific target groups), which manifests as increased and sustained vaccine demand, improved vaccination rates in those specific groups, and perhaps also the expansion of programs to other groups, can drive higher volumes of seasonal vaccine manufacture, which is a key factor in pandemic capacity. Indeed, recent decreases in seasonal influenza VCRs in HICs represent a threat to manufacturers’ ability to be ready to respond to the next influenza pandemic [22].

### 2.3. Economic Incentives Are Crucial for Mobilizing Private Investment and Expertise in Pandemic Planning During the Inter-Pandemic Period

Public funding is essential for the development of pandemic influenza vaccines because there is effectively no commercial market for these products. When companies are making investment decisions about their portfolios and manufacturing facilities, this lack of certainty can play a role in choosing not to invest in pandemic influenza vaccines. There are, however, incentives that are deployed to varying extents:

- **APAs:** These guarantee a purchaser, reducing financial risk for manufacturers and potentially incentivizing investment in R&D. Such agreements provide a safety net for vaccine manufacturers by ensuring demand and covering some of the financial risks inherent in scaling up production (bulk antigen and fill and finish) for a product that may not ultimately be needed in large quantities if a pandemic does not materialize. As such, these commitments are a proven economic incentive that aligns vaccine industry innovation with public health needs. By offering demand clarity and reducing financial risks, APAs encourage vaccine development and can ensure that manufacturers are ready to respond rapidly and equitably when the next pandemic emerges.
- **Public procurement contracts:** Large, upfront purchases (e.g., stockpiles of pre-pandemic vaccines, adjuvants and/or critical vaccine components such as syringes, vials, and egg supply) by governments provide predictable revenue streams, encouraging companies to prioritize pandemic vaccine development.
- **Tax incentives and grants:** Direct financial support for research, infrastructure (such as construction and/or upscaling of manufacturing facilities), and clinical trials lowers the cost barrier for pandemic influenza innovation. Several OECD Governments use various tax credit schemes allowing companies to recoup a significant percentage of certain research expenditures to offset the high costs and risks of developing new vaccines and technologies. Until early 2025, direct grants from organizations such as NIAID/NIH, BARDA, CEPI, and the Flu Lab have been funding research and novel vaccine development, support clinical trials, and helping to expand manufacturing capabilities for influenza countermeasures. However, post-COVID fiscal constraints and shifting political priorities have cast uncertainty on sustained public funding from the US, which was historically the strongest public funder of influenza vaccine innovation.

Here are three strong examples of successful national pandemic planning involving the integration of the private sector: The US Department of Health and Human Services (HHS) and its agencies (FDA, BARDA, CDC) have developed comprehensive plans that explicitly assign roles and responsibilities to private sector stakeholders, ensuring continuity of operations and coordinated response [23]. In 2021, Canada released its Biomanufacturing and Life Science Strategy (BLSS) to advance pandemic readiness by strengthening Canada’s domestic capacity to produce vaccines, therapeutics, and other countermeasures. The Health Emergency Preparedness and Response Authority (HERA), formed in 2021 under the European Commission, established cooperation and coordination working groups that involve private vaccine manufacturers in preparing for responses to threats and securing access to vaccines and countermeasures. Figure 1 shows how pandemic preparedness is anchored on seasonal vaccine programs.

Since these mechanisms can be challenging for LMICs at a national level, the model deployed during the COVID-19 pandemic by the COVAX alliance should be considered for future influenza pandemic planning. The lessons from COVAX’s interventions during COVD-19 are largely documented elsewhere; however, the Advanced Market Commitment (AMC) model—with adaptations restricted to LMICs, for example—could be effective in bridging the manufacturers allocations committed to WHO (see Section 4.2 PIP Framework) to the likely larger LMIC needs. AMCs could better prime the interface with manufacturers ahead of a future pandemic, and permit the committed volumes to be included in equitable global preparedness planning.

### 2.4. Diversifying Vaccine Platforms Is a Risk Mitigation Strategy

Developing a vaccine is a resource-intensive process—with estimates ranging from USD 31–68 m for early stages to nearly USD 900 m for full development and licensure [24]—that involves significant scientific and financial risks. For companies that innovate in the influenza space, this innovation is generally directed towards improving seasonal influenza vaccine performance (duration, breadth, amplitude of response). Such investment can have positive knock-on effects in developing a pandemic response vaccine, but historically, public funding has been the primary driver for vaccine companies to invest in pandemic-ready platforms. The US Biomedical Advance Research and Development Authority (BARDA) provided upfront capital for clinical development and manufacturing scale-up, de-risking investments in technologies such as adjuvants, cell culture, recombinant, viral vector platforms, and new delivery methods. Until recently, BARDA was the principal funder of the mRNA platform research and development of viral vaccines; and this investment was leveraged during the COVID-19 pandemic. The recently announced cuts to funding, if maintained, will harm influenza vaccine innovation and therefore pandemic readiness.

In the short- to mid-term, the innovations that will advance pandemic preparedness are essentially:

- **mRNA/SAM (self-amplifying mRNA) vaccines:** Pending the outcome of ongoing clinical trials, the main feature of this technology is speed of response and scale-up.
- **Cell-based and recombinant vaccines:** These platforms offer long-standing safety records with real world experience spanning several years and age groups.
- **Adjuvants:** Adjuvants such as MF59 and AS03 will play a significant role in antigen-sparing and hence, manufacturing capacity optimization.
- **Delivery systems:** While intranasal sprays (using live attenuated strains) are likely to be used in a future pandemic, microneedle patches and oral formulations require further work to demonstrate their improved immunogenicity and ease of administration, and the latter is highly advantageous in a pandemic setting.
- **Cold chain independent vaccines:** Developing vaccine platforms or formulations that are stable at higher temperatures reduces logistical barriers, especially in Low- and Middle-Income Countries (LMICs).

Portfolio diversification not only mitigates risks but also expands the range of tools available for pandemic response; this benefits both private and public entities and increases the likelihood of equitable access to effective vaccines. 

## 3. Leveraging Regional Engagement to Improve Equity

### 3.1. Export Restrictions Enhance Existing Supply Chain Vulnerabilities

The COVID-19 pandemic highlighted the fragility of global supply chains and the risks posed by export restrictions. During the crisis, 104 countries imposed bans or limits on the export of vaccines and critical raw materials, aiming to secure domestic access but inadvertently undermining global production and equitable distribution. Major producers—including the US, the EU, and India—implemented measures ranging from formal export authorizations to informal prioritization of domestic needs, often justified by national emergency frameworks or fulfillment of APAs with manufacturers. These restrictions disrupted the complex, interdependent supply networks required for vaccine manufacturing, which rely on hundreds of components sourced internationally. Supply chain vulnerabilities are exacerbated by two factors:

**Concentration of manufacturing**: A handful of countries dominate global vaccine production, making the system susceptible to bottlenecks and geopolitical tensions. As of 2024, there are about 30 bulk vaccine manufacturers worldwide and 12 companies that only perform fill and finish operations, collectively responsible for producing 900 million trivalent-equivalent doses annually. The largest concentrations of manufacturing sites are in Europe and Asia, with notable sites in North America, Australia, and Latin America

Brazil stands out as an MIC where domestic manufacturing was established through a Production Development Partnership (PDP), with a state vaccine manufacturer, the Butantan Institute, that partnered with French company Sanofi to enable the production of trivalent influenza vaccine doses. Butantan increased its production to accommodate more at-risk groups, increased seasonal vaccine demand, and to prepare for an influenza pandemic [25]. Vietnam established domestic seasonal and pandemic influenza vaccine production through the Institute of Vaccines and Medical Biologicals (IVAC), culminating in licensure of locally made vaccines in 2019 [26]. National production lowered vaccine costs and increased public trust, enabling broader access; however, overall influenza vaccine uptake remains low due to limited awareness, with significant differences between urban and rural areas [27]. Other countries such as Mexico, China, and India have built manufacturing capabilities to meet their program needs, though vaccination coverage varies across these settings. In South Africa, the Biovac Institute, a biopharmaceutical company founded in 2003 as a public–private partnership with the government, plays a central role in domestic vaccine manufacturing efforts, including influenza and other pandemic threats. Biovac established partnerships with Sanofi and Pfizer, enabling technology transfer and local production of several vaccines. Initiatives such as the mRNA Vaccine Technology Transfer Hub (WHO-led), centered around a collaboration between Afrigen Biologics, Biovac, and the South African Medical Research Council (SAMRC), further illustrate cross-collaborations designed to scale up regional preparedness for pandemic response. Notwithstanding these positive examples, there are currently no bulk influenza vaccine production facilities in the African region. A majority of LMICs rely on vaccine imports [5].

**Dependence on complex, globalized supply networks:** During a pandemic, important disruptions may occur because a pandemic influenza strain will emerge suddenly and spread rapidly, requiring manufacturers to adapt their production processes quickly and scale-up output, starting from scratch with a novel strain and under severe time pressure. Delays or breakdowns anywhere in the supply network—whether caused by disruptions in sourcing raw materials, regulatory bottlenecks, or logistical hurdles—can lead to major shortages and inequities in vaccine distribution.

Since the government export restrictions during COVID-19 were a response to intense political pressure, they are likely to recur, and ensuring upfront, explicit government commitment that can be communicated transparently could mitigate that future risk to some extent.

### 3.2. Diversified Supply Security and Capacity Building Will Improve Equitable Access

There is growing momentum toward regionalization of vaccine manufacturing, i.e., distribution across multiple geographical locations, versus its concentration in a few high-income countries, and skilled workforces are a key component of success. Regionalization would not only enhance supply security but also support local economic development and capacity building.

Establishing regional manufacturing hubs in Africa, Latin America, Asia, and other underserved regions can reduce dependence on a few global suppliers and support local economic development and capacity building, improving resilience. Partnerships like the African CDC’s Partnerships for African Manufacturing (PAVM), aiming to produce 60% of Africa’s vaccines locally by 2040, could reduce reliance on global supply chains. Launched in 2022 by the World Economic Forum, the US National Academy of Medicine, and CEPI, the Regionalized Vaccine Manufacturing Collaborative (RVMC) is the only organization dedicated exclusively to advancing the vaccine regionalization agenda [28]. These initiatives aim to build sustainable, scalable capacity that can serve both routine immunization needs and surge demands during health emergencies. However, these efforts face challenges of scale, financing, and technical expertise, and must be coordinated to ensure they complement rather than fragment the global vaccine ecosystem.

Multinational companies regularly engage in technology transfer agreements with local manufacturers; such partnerships may or may not be supported by public or philanthropic funding, or geopolitical political interests. Equitable access is also a key driver for these projects. Since manufacturing processes may not be easy to transfer in an emergency, preparation through the creation of infrastructure and trained staff ahead of time are essential for a timely pandemic response. This approach is not new. The WHO Global Action Plan for Influenza Vaccines (GAP), launched in 2006, showed that long-term national commitment, clear vaccination policies, and year-round demand are the key to maintaining viable manufacturing and enabling a rapid pandemic response [29]. Fourteen manufacturers (public and private) partook across 14 MICs, producing seasonal and/or pandemic strain vaccines. Although the results were mixed [30], they showed that long-term vision was critical for success, with a strategic and holistic approach by the national government being a key contributor, including a seasonal vaccination policy with clear target groups and market access in place. Domestic manufacture may enable the changeover to pandemic strains as part of a country/region’s pandemic response, assuming a similar manufacturing process.

If the renewed interest in regional hubs by RVMC post COVID-19 is successful in deploying an economically viable model, i.e., sustained manufacture and supply during inter-pandemic periods to the region (or beyond) through national and/or pooled tenders, then they could significantly mitigate the inequities seen during COVID-19. 

When discussing technology transfer, particularly for new manufacturing technologies, it is also important to mention Intellectual Property and the associated waivers. Perceived as a potential strategy to improve vaccine equity, they would in fact not solve production and distribution complexities inherent to vaccine manufacturing [31]. Some companies have entered into voluntary licensing agreements with third parties to facilitate the creation of a broader manufacturing footprint. This can be an important first step, with the keyword here for manufacturers being “voluntary”.

Pre-negotiated export waivers are advance agreements between countries or within trade blocks to exempt certain essential goods from national export restrictions during emergencies like a pandemic. Such mechanisms reduce border delays, support global supply chains, and ensure that shortages in one country do not cripple collective pandemic response capabilities. A recent example was during the COVID-19 pandemic; the US adapted its export control rules for certain medical supplies, most notably for exports to neighboring countries like Canada and Mexico. The US and the EU periodically updated their lists of restricted and exempt goods, issuing waivers for key medical products as the crisis evolved. However, the US imposed restrictions on the export of key raw materials for the manufacture of COVID-19 vaccines, leveraging the Defense Production Act (DPA) and prioritizing domestic production. Similarly, the EU adopted a regulation which only authorized the export of vaccines against COVID-19 if the “volume of exports to specific countries is not such that it poses a threat to the execution of the EU APAs”. India, after initially exporting COVID-19 vaccines globally, restricted these exports when the pandemic surged domestically, reducing vaccine access in LMICs [32]. Strengthened World Trade Organization (WTO) involvement could help formalize export waivers, enabling more reliable vaccine distribution during pandemics.

### 3.3. More Efficient Access Can Be Achieved with Collaborative Regulatory Processes

Diverse and sometimes conflicting regulatory requirements across countries slow down clinical trials, approvals, and distribution, making it harder for vaccine developers to scale-up production with confidence and deploy new vaccines quickly during health emergencies.

Regional empowerment in regulatory assessments can bring efficiency and pragmatism, meaning quicker reviews and approvals and should not be perceived as fragmenting competencies. Only 30% of LMICs have regulatory agencies capable of approving novel influenza vaccines. Considerable effort has been put into achieving sovereign regulatory decision-making capacity building by WHO and the Gates Foundation. For influenza, specifically, WHO’s preparedness plan targets high-performing regulatory systems within countries and earmarks part of their budget for this. Not only does this targeted evolution go well beyond influenza, but we also question how realistic this output is. Additionally, during a pandemic, multiple parallel assessments of regulatory dossiers for vaccines or other products constitute a huge workload for the manufacturers’ regulatory and technical teams; some country-specific requirements are contradictory and can even slow product release. Alternatives, specifically using reliance models to facilitate emergency use approvals and inspections based on bona fide reports from trusted mature regulatory authorities, accelerate access and optimize the use of the combined knowledge pool during a pandemic crisis and should be preferred. There is evidence of successful navigation, for example, by Brazil’s ANVISA during COVID-19 [33]. In practice, half of the 48 countries receiving PIP PC funding used a collaborative approach for COVID-19 vaccine approval. This is a great baseline to build upon. Reliance or collaborative approaches as best practices could be prospectively embedded in national preparedness plans during the inter-pandemic period. We suggest that efficiency gains be directed towards improving domestic pharmacovigilance capacity, with the virtuous knock-on effect of increasing population confidence in safety reporting and transparency, and contributing to vaccine confidence, which is a critical component of a successful pandemic response.

## 4. It Is Crucial to Strike a Balance When Considering Vaccine Pricing and Available Vaccine Supplies

Prices must be affordable enough to promote widespread government programs, but also sufficient to ensure stable supply and the continued capital investment by manufacturers that is necessary to maintain compliance with quality standards.

### 4.1. Demand-Based Tiered Pricing

The price of seasonal influenza vaccines varies significantly across regions. The Pan American Health Organization (PAHO) Revolving Fund centrally procures seasonal influenza vaccines, for which agreed prices are based on a pooled demand from up to 17 countries in the region. This allows vaccine manufacturers to receive predictable numbers of doses that they can match with their supply plan. We believe that one of the most effective tools for achieving more readily equitable access to seasonal influenza vaccines in LICs is demand-based tiered pricing, with countries potentially adopting pooled demand/procurement mechanisms, and with the option of spanning several years, supporting predictability. In practice: 

HICs: These countries pay higher prices per dose, reflecting their greater ability to pay and helping to subsidize the costs of R&D and manufacturing. HIC governments often provide most of the funding for pandemic influenza vaccine innovation, infrastructure, and clinical trials. This cross-subsidization allows vaccine suppliers to recoup the cost of R&D and capital expenditure with a higher price while offering lower prices to LMICs.

LMICs: These countries benefit from lower, sometimes even at-cost pricing, ensuring affordability is not a barrier to access. 

Tiered pricing has been successfully implemented for other vaccines (PAHO, Gavi) and medicines (Global Fund, PEPFAR) and may increasingly be adopted for pandemic vaccines. Organizations like Gavi pool resources from donor countries to negotiate lower prices and secure supply for LMICs. This is how, during the COVID-19 pandemic, COVAX enabled rapid scaling of vaccine production and distribution of innovative vaccines to LMICs, demonstrating their effectiveness in a global crisis. COVAX enabled advanced purchases and equitable distribution of over 1.8 billion COVID-19 vaccine doses to 87 LMICs [34]. This model aligns commercial incentives with public health goals, ensuring that innovation is rewarded while access is broadened. 

### 4.2. Multilateral Frameworks and Accountability as a Lever for Increased Equity

Created by WHO in 2011, the principal multilateral framework for pandemic influenza is the Pandemic Influenza Preparedness (PIP) Framework (FW) for the sharing of influenza viruses and access to vaccines and other benefits [35]. Amongst other matters, the PIP FW stipulates how pandemic influenza manufacturers contribute both financially and in kind (vaccine doses) to the preparation of a future influenza pandemic. In return, they access the viral strains to develop future pandemic vaccines, like for H5N1. Seasonal and pandemic influenza vaccine manufacturing are intrinsically linked in this unique Access and Benefit Sharing system, since payments to WHO by manufacturers are based on seasonal influenza vaccine sales. Whilst not obligatory, the majority of manufacturers do pay. The payments go into a fund called the Partnership Contribution (PC). Contributions can reach millions of dollars for companies with substantial seasonal influenza vaccine sales. WHO, along with a PIP Advisory Group, develops six-year strategic plans called High Level Implementation Plans (HLIPs) to guide how this money is spent [36]. Although the private sector, as “partners,” is invited to comment on draft plans, there is a perception that comments and requests are not sufficiently considered. This can result in a level of frustration around the spending of the PC since, despite reporting being overseen by the PIP Secretariat at WHO, concrete progress can be difficult to interpret in terms of true preparedness [37].

In terms of equity, the PIP FW does mean that for the 15 participating manufacturers who have signed a binding contract with WHO, a percentage of their pandemic volume would be attributed in real time to WHO. Depending on the manufacturer, this can be up to 15% of the total being produced, provided as a combination of donations and low prices [38] in parallel with higher incomes purchasing directly from the manufacturer. The principle is highly equitable; however, its feasibility has never been tested. The ability to export up to 15% of vaccine volume in real time from the country of manufacture (e.g., India, US, South Korea, China) to LICs under the auspices of the PIP FW and WHO oversights remains a question mark. And while childhood program vaccinations are routine in many LICs, thanks to Gavi and its partners (including WHO in-country staff and UNICEF), supporting the infrastructure required to immunize adults remains a challenge [39]. This underlines the utility of establishing sustained seasonal influenza programs, particularly in HCWs and other vulnerable adults. Programs can be leveraged to prepare at least certain segments of the adult population to be more easily reached when a pandemic vaccine is deployed. Table 1 provides a synthesis of our analysis of the PIP FW strengths, limitations, and suggestions for potential evolutions.

Overlaid on this backdrop is the agreed text of the Pandemic Agreement. Article 12 of the Agreement outlines a pathogen access and benefits system modeled on the PIP FW [40]. It is likely that at some point in the future, in the interests of efficiency, influenza may be rolled into this global pandemic preparedness framework, and the PIP FW disappears. Replication of the PIP model, which the private sector does not consider to be fit for purpose, is a bold move and is not without risk. We emphasize therefore that lessons must be learned from analyzing PIP feedback from manufacturers, both individually and via their respective trade associations, and carefully taken into account during the implementation phase of the Agreement. This implementation must embed an interface with industry that gives equal credence to manufacturer input alongside the public sector; acquiring buy-in through shared ownership is much more likely to result in commitments being fulfilled in due course.

Lastly, and importantly, rigorous transparency and meaningful, realistic (versus aspirational), simple metrics are factors that drive sustained private sector engagement, as well as government funding. The Gavi public–private partnership model that endeavors to “combine the technical expertise of the development community with the business know-how of private sector” could be investigated as part of improving the current interface. Indeed, a recent MOPAN assessment rated the organization “satisfactory” on 12 out of 12 Key Performance Indicators that span sustainability, partnerships, and cost-consciousness financial transparency [41].

## 5. Aligning Private Sector Incentives in Achieving Equitable Pandemic Preparedness

Pandemic influenza preparedness is a complex and technical subject; our discussion here is limited to areas where the interface between the private and public sector can be improved as both work towards a common goal. Table 2 outlines some of the respective roles of private manufacturers and governments, including multilateral actors such as WHO and NGOs, across key domains, looking at areas that could benefit from more concrete collaboration. We have summarized some solutions that we believe merit further work, under the auspices of a robust PPP, to develop measurable, realistic targets and timelines.

## 6. Conclusions

Pandemic influenza preparedness begins with building and sustaining a strong supply and demand for seasonal influenza vaccines. Furthermore, true pandemic influenza preparedness requires a deeper partnership between the vaccine-developing private sector and global health governance. The private sector’s capacity for innovation and rapid scale-up is indispensable but must be harnessed within frameworks that provide at least partial finance for investment in research and development to sustain the prioritization by the private entity faced with competing non-pandemic projects. Economic incentives such as APAs and tiered pricing can align commercial imperatives with public health goals; however, they must come with appropriate accountability too. Regionalization including technology transfer in collaboration with the private sector can be a strong path to equitable, timely availability of pandemic vaccines; it must be undertaken voluntarily and be part of a holistic immunization strategy for the country and/or region to be meaningful across time. The importance of having the private sector at the table in pandemic influenza discussions at a global level is underlined. A dialog that builds an appropriate level of mutual trust and understanding of respective challenges and obligations is essential to establish ahead of the next influenza pandemic.


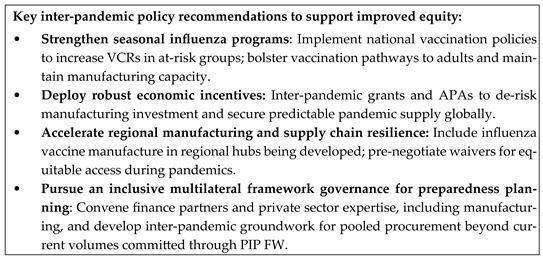


### Limitations

While this perspective identifies certain strategic priorities for pandemic influenza preparedness, it is important to acknowledge several limitations. The analysis is shaped by the authors’ experiences and available data, which may not capture all global and local challenges. Many countries, especially LMICs, face significant barriers—constrained health and laboratory infrastructure, uneven utilization of available funding, staff shortages, and supply chain disruptions—that may impede effective and timely preparedness. Data on the cost-effectiveness and scalability of the proposed solutions, such as seasonal influenza programs and technology transfer, and long-term positive outcomes of APAs (especially in LMICs) remain limited. This perspective is constrained by the unpredictability of future pandemic threats and by fluctuating political will, funding priorities, and the logistical feasibility of sustained international cooperation. Therefore, while strategic industry engagement and improved policy frameworks are essential, future analyses should address the operational gaps and complexities to ensure recommendations can be adapted for diverse global contexts.

Readers are encouraged to consider this perspective as a contribution to ongoing discussion, and to consult additional sources. Continued dialog and collaboration are essential to address outstanding challenges and to adapt strategies for diverse settings and emerging threats.

## Figures and Tables

**Table 1:** PIP FW: Summary of key strengths, limitations, and potential evolutions.

Strengths	Limitations	Potential Evolutions
Pre-arranged legally binding commitments “SMTA2” with 15 manufacturers.	Applies only to existing manufacturers who have voluntarily made a commitment. Not all vaccines are pre-qualified; this may slow country use.	Improved inclusivity (antiviral and diagnostic manufacturers). Development of a rapid process to add “new” manufacturers that may emerge in response to a pandemic. WHO pre-qualification for all products.
Predefined volumes determined for real-time supply to WHO.	Estimated volume of approximately 700 million doses for LMICs could fall short of country needs.	Broaden participating vaccine manufacturers. Leverage alternative mechanisms to secure more volume, e.g., “Covax- like” AMC.
Framework in place with agreements signed by manufacturers.	Practicalities of the 2011 FW are untested, inter alia, the ability to export vaccine from the countries of manufacture during a pandemic.	Obtain upfront government commitments to allow timely exports from countries with manufacturing. Run joint pilot drills with selected entities to identify and remediate bottlenecks.
Constitution of a fund (PC) to support global- and country-level pandemic preparedness and implementation through HLIPs	The PC relies on a limited number of manufacturers’ voluntary payments, which may not be a sustainable funding source. PC spending assigned to topics beyond influenza, e.g., Infodemics.	Diversification of sources of funding; involvement of multilateral development banks. Confine PC spending to improving pandemic influenza preparedness specifically.
Interface between WHO, countries, civil society, and manufacturing industry is established; governance in place.	Collaboration could be optimized.	Evolve towards a public–private partnership model to improve share of voice for all contributors to preparedness. Set up appropriate transparency mechanisms for public and private investments.

**Table 2:** Improving interfaces between private manufacturers and the public sector in pandemic preparedness.

Areas That Would Benefit	Private Sector Roles	Public Sector (Governments)/WHO Roles	Identified Solutions
Seasonal vaccine demand generation	Increasing manufacturing capacity based on increased demand for seasonal and therefore pandemic vaccines.	Seasonal influenza program policy implementation in countries. Delivery programs to adults (e.g., healthcare workers, vulnerable elderly people, pregnant women).	Apply realistic metrics using Key Performance Indicators (KPIs) to track progress, e.g., seasonal VCRs in HCW ≥ 60% within x years.
R&D for pandemic vaccine candidates	Resource allocation and financial support. Clinical study design and conduct, regulatory strategy expertise. New cutting-edge antigen platforms.	De-risking through co-financing; provision of tax incentives. Oversight on programmatic suitability.	Broad adoption of collaborative regulatory procedures
Manufacturing footprint	Anticipate surge capacity particularly for fill and finish activities. Voluntary Technology transfer to suitable partners preferably in the inter-pandemic period.	Continued support for “local” manufacturers. National campaigns promoting seasonal influenza programs. Regulatory support to maintain WHO pre-qualified product status.	Regional/national hubs with activity linked to government policy and program implementation for sustainability.
Supply Chain Resilience	Stockpiling of critical components. Local partnerships.	Export waivers (for pandemic supplies). Cold chain supply and storage mapping.	Resiliency assessment process of end-to-end network
Inter-pandemic vaccine supply planning	Vaccine platform diversification. APAs. Permanent state of readiness (across all involved functions). Demand-based tiered pricing policy. Provision of the pre-agreed volume %to WHO (per PIP FW).	Binding vaccine volume commitments, APA framework development, pandemic influenza procurement, storage and distribution plan (as part of national plans).	Transparent allocation systems. Prioritization of secure pandemic supply plans. Delivery pathways for vaccination (especially adults). Pooled procurement mechanism for pandemic vaccine purchase.
Public—private interface	Provision of expertise into global frameworks. Real world experience of vaccine distribution at scale.	Oversight of the Framework’s implementation. Coordination across functions (e.g., Emergency Use Licensures). Scenario planning (vaccine demand). Contractual compliance.	Establish a PPP that will draw expertise from all sectors to ensure the Framework for a future influenza pandemic is realistic and achievable by all stakeholders.

## Abbreviations

The following abbreviations are used in this manuscript:

WHO	World Health Organization
APA	Advance Purchase Agreement
AMC	Advanced Market Commitment
US	United States of America
VCR	Vaccine Coverage Rate
WHA	World Health Assembly
TB	Tuberculosis
HIV	Human Immunodeficiency Virus
NCD	Non-Communicable Disease
PIVI	Partnership for International Vaccine Initiatives
PPP	Public–Private Partnership
IVAC	Institute of Vaccine and Medical Biologicals
PDP	Product Development Partnership
OECD	Organization for Economic Cooperation and Development
BARDA	Biomedical Advanced Research and Development Authority
CEPI	Coalition for Epidemic Preparedness Innovations
NIAID/NIH	National Institute of Allergy and Infectious Disease/ National Institute for Health
HHS	Health and Human Services
FDA	Food and Drug Administration
CDC	Center for Disease Control
BLSS	Biomanufacturing and Life Science Strategy
HERA	Health Emergency Preparedness and Response Authority
H/L/MIC	High/Middle/Low Income Country
RVMC	Regional Vaccine Manufacturing Collaborative
PAVM	Partnerships for African Manufacturing
DPA	Defense Prevention Act
WTO	World Trade Organization
PIP	Pandemic Influenza Preparedness
PC	Partnership Contribution
ANVISA	Brazilian Health Regulatory Agency
PAHO	Pan American Health Organization
GISRS	Global Influenza Surveillance Response System
HLIP	High Level Implementation Plan
HCW	Healthcare Workers
SAMRC	South African Medical Research Council
KPI	Key Performance Indicator
